# Nonconformity and Medicine

**Published:** 1910-07-16

**Authors:** 


### Editor's Letter-Box.

### NONCONFORMITY AND MEDICINE.

*To the Editor of* The Hospital.

Sir,—"J. M. S." has made a mistake. It was not medical nonconformists who were remonstrated with, but lay nonconformists like Mr. W. T. Stead, and those only when they show so patent a bias in favour of the irregular medical practitioner. For the rest, probably "J. M. S." and the undersigned have more opinions in common than the former seems to suppose. But a medical journal is, of course, neutral on all non-medical questions. It has to preserve balance; to remember that many sincere people think differently on the subjects he mentions. Religion, politics, advanced humanitarianism, social customs—here is a pretty assortment of red rags! So "Who that pretender is, and who that king," the medical journalist has no right to assert.

Yours faithfully,
The Writer of the Note.

